# Effective doses received by the gastrointestinal tract compartments of adults due to food intake in Egypt

**DOI:** 10.1038/s41598-025-86291-6

**Published:** 2025-01-28

**Authors:** Yasmine Abdalbasit, Khaled Salahel Din, Abdelbaset Abbady, Nagwa Saad

**Affiliations:** 1 Physics Department, Faculty of Science, South Valley University, Qena, 83523 Egypt; 2 Faculty of Computer and Information, South Valley University, Qena, 83523 Egypt

**Keywords:** Natural radionuclides, (HPGe) detector, GIT model, Effective dose, Biophysics, Physics

## Abstract

^226^Ra, ^232^Th, and ^40^K levels in various foods frequently consumed by Egyptians were determined using a gamma-ray spectrometer based on the germanium detector (HPGe). Activity concentrations of ^226^Ra, ^232^Th, and ^40^K were in the range of < 0.10 to 0.79 ± 0.07, < 0.09 to 0.42 ± 0.04, and < 1.96 to 89.73 ± 2.96 Bq/kg, respectively. The gastrointestinal tract (GIT) model was employed to estimate the effective doses received by the different parts of the adult’s gastrointestinal tract, i.e., stomach (ST), small intestine (SI), upper large intestine (ULI), and lower large intestine (LLI), due to the ingestion of the analyzed foods. This estimation was based on mathematical calculations of the energy absorbed by organs due to transformations of ingested radionuclides. The effective doses (μSv/y) received by each compartment were 8.86 (ST), 8.76 (SI), 66.90 (ULI), and 176.76 (LLI). The results do not exceed the safe thresholds set by global organizations UNSCEAR and WHO, 290 and 250–400 μSv/y, respectively. Therefore, radionuclide intakes due to investigated food consumption do not pose any significant radiological impact.

## Introduction

Natural radioactivity is omnipresent in the environment, with the majority of human radiation exposure deriving from cosmic and primordial sources. Although guidelines often emphasize artificial radiation sources^1^, radionuclides naturally occur in food and water, as plants absorb them from soil during their growth. Consequently, radionuclides are found in plant- and animal-derived foods^2^, and they can build up in fish in aquatic life. The concentration of radionuclides in consumables varies with local geology, climate, and agricultural methods. Despite typically low levels, natural radionuclides can pose health risks upon ingestion or inhalation, as they decay within the body over time and emit energy absorbed by body tissues^3,4^, which can damage tissues and cells^5,6^. Therefore, understanding natural radiation’s impact on biological tissues is vital for assessing long-term radiation exposure and formulating public health safety measures. Studying natural radionuclide levels in food aims to assess their impact on human health^7^. Key factors evaluated usually include radiation absorbed dose rate, annual effective doses, and lifetime cancer risk based on food consumption rates and constants specific to these radionuclides^8–10^.

Biokinetic models provided by the International Commission on Radiological Protection (ICRP) can calculate organ-absorbed doses. These mathematical models simulate radionuclide behavior after ingestion, inhalation, or blood absorption, considering radionuclide type, quantity, bioavailability, distribution, excretion, and energy absorption resulting from their nuclear transformations, enhancing the understanding of radiological effects and ensuring compliance with dose limits in scientific applications^11–13^.

A previous study using ICRP biokinetic model found key target organs for uranium retention to be the kidney, liver, non-exchangeable bone volume, and soft tissues^14^. A study evaluated annual organ-equivalent doses due to the ingestion of food crops using the Radiation Toolbox software that is based on a biokinetic model and found that 40K’s annual organ-equivalent dose in the large intestine is higher than in other organs, while the doses from ^238^U, ^226^Ra, ^210^Pb, and ^232^Th were primarily at the bone surface^15^. While a study calculated the retention and committed doses in the gastrointestinal tract after 1 h of ingestion of 1 Bq of ^226^Ra in various age groups using IRDA software, showed the highest committed equivalent dose in the lower large intestine (LLI) (8.32 μSv for adult males, higher for 1-year-olds)^16^. A similar study stated that ingestion of ^60^Co caused a maximum dose in LLI at 9.44 × 10^−3^ μSv for adult males^17^. Using the ICRP biokinetic model, studies found that effective doses for the adults due to ingestion of table oil containing ^238^U, ^232^Th, and ^222^Rn reached 11.6 ± 0.7 µSv/y^18^. And due to the ingestion of foodstuffs containing ^238^U and ^232^Th, the effective doses are 33.2 and 128 µSv/y, respectively^19^. Also, ingestion of olive oil yielded a maximum committed effective dose of 5.9 μSv/y, and the committed effective dose to skin from olive oil masks was found to be 0.07 mSv/y cm^2^ for adult members of Morocco^20^. A mathematically calculated annual dose equivalents to the whole body from ingestion and inhalation of 26 Bq of ^40^K in Mexican tobacco were found to be 0.23 µSv and 15.8 µSv, respectively^21^.

**Fig.1:**
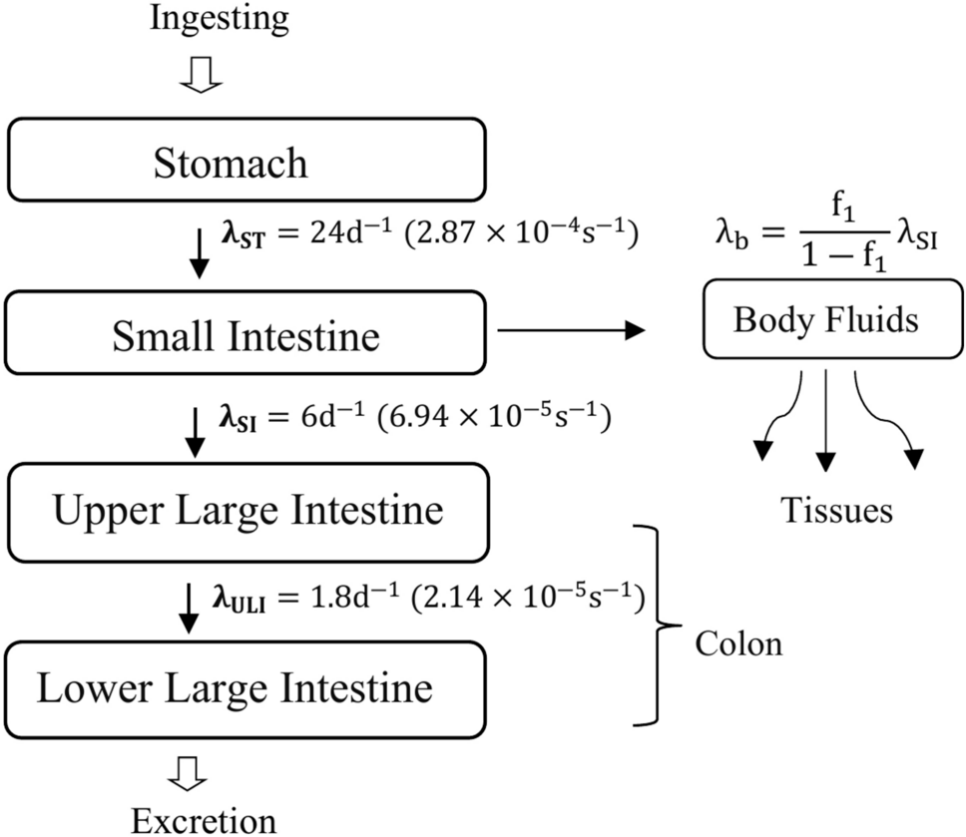
Model for the gastrointestinal tract (GIT).

The study employs bio-modelling of the gastrointestinal tract (GIT Model) to mathematically calculate the effective doses absorbed (μSv/y) by each of the four GT compartments separately of the adult people due to the ingestion of natural radionuclides. Based on ICRP-30 recommendations^16^, the model represents the GT as four compartments: stomach (ST), small intestine (SI), upper large intestine (ULI), and lower large intestine (LLI) (Fig. 1) and simulates the translocation and digestion behavior of radionuclides through GT compartments at varying rates according to their physical and chemical properties. ST is the entry point for radionuclides, some of which are absorbed into the bloodstream through the walls of SI and eventually expelled via LLI in faces^22,23^. The radionuclides can irradiate both the digestive contents and the wall (tissue layers, including mucous, and muscular) of each compartment^24^. The present study aims to assess the internal radiation dose for each GIT compartment separately, considering each compartment as a source and/or target organ.

## Materials and methods

Fifty-one food and beverage samples were gathered in the period of 2022–2023 from local Egyptian markets to represent commonly consumed items, particularly those not extensively studied before, like soft drinks and instant noodles. The samples were categorized into five groups: beverages (17 samples), processed cereals (18 samples), milk/dairy products (6 samples), fish/meat products (6 samples), and table salt (4 samples). Beverage samples were filled directly into the Marinelli beaker (~ 700 ml), while tea and powdered milk were prepared in the same manner as they used for drinking purposes using distilled water. Processed cereals and table salt samples were first dried and ground into powder before being filled in the Marinelli beakers. Dairy products were prepared without any treatment and filled directly, while fish/meat products were first mixed using a grinder until they formed a homogeneous dough and then filled into the Marinelli beaker. Samples were then stored in the refrigerator. All samples were stored in the sealed polypropylene Marinelli beakers (GA-MA Beakers 590G-E) for four weeks to reach secular equilibrium between long-lived radionuclides and their short-lived progenies^25^ before analysis.

The activity concentrations of ^226^Ra, ^232^Th, and ^40^K in the prepared samples are measured using an N-type HPGe (CANBERRA) detector encased in a 100 mm thick cylindrical lead shield, cooled with liquid nitrogen, coupled with a computer-based multi-channel analyzer with a 40% relative efficiency, 16,000-channel spectrum memory, detector’s spectroscopy capabilities range from 0 up to 3 MeV, and 2 keV energy resolution at 1332 keV gamma-ray energy of ^60^Co^26^. The system’s efficiency was calibrated using LABSOCS efficiency calibration software, with peak efficiency mathematically represented as a function of energy utilizing detector characterization, geometry templates, sample parameters, and reference material (plant IAEA-330) was used to ensure the accuracy of the theoretical calibration. Genie 2000 software was used for spectrum collection and analysis involving a 72-h measurement time for both sample and a background. Peak areas in the gamma-ray spectrum are calculated to determine the number of decaying radioactive atoms in the sample during the measurement time^27^.

The activity of ^226^Ra was gauged using gamma-ray lines of 295 and 351 keV from ^214^Pb as well as 609 and 1120 keV from ^214^Bi. For ^232^Th, gamma-ray lines of 238.63 keV from ^212^Pb, 583.2 and 2614 keV from ^208^Tl, and 911 keV from ^228^Ac were used. The activity of ^40^K was directly measured by its gamma emission at 1460.8 keV^28^. The concentrations of ^226^Ra, ^232^Th, and ^40^K activities in Bq/L or Bq/kg for the samples were calculated with the given Eq. (1)^29^.

1 $${\text{A }} = \frac{{\text{N}}}{{ \upvarepsilon_{{\left( {{\text{E}}\upgamma } \right) }} \times {\text{t}}_{{\text{c }}} \times {\text{I}}_{{\upgamma \left( {{\text{E}}\upgamma } \right) }} \times {\text{M}}}}$$

where N is the number of counts in each peak area corrected from the background at a given energy E, ε is the detection efficiency at energy E, M is the mass/volume (kg or L) of the measured sample, t_c_ is the counting lifetime, and I_γ_ is the number of gammas per disintegration.

The uncertainty of the activity concentration (UA) was calculated taking into account sample count uncertainty (UNs), background count uncertainty (UNB), detector efficiency uncertainty (U$\upvarepsilon_{\upgamma }$), and sample mass uncertainty (Um) according to the following formula Eq. (2)^30^.

2 $${\text{U}}_{{\text{A}}} = \sqrt {\left( {\frac{{{\text{UN}}_{{\text{S}}} }}{{{\text{N}}_{{\text{S}}} }}} \right)^{2} + \left( {\frac{{{\text{UN}}_{{\text{B}}} }}{{{\text{N}}_{{\text{B}}} }}} \right)^{2} + \left( {\frac{{{\text{U}}\upvarepsilon_{\upgamma } }}{{\upvarepsilon_{\upgamma } }}} \right)^{2} + \left( {\frac{{{\text{U}}_{{\text{m}}} }}{{\text{m}}}} \right)^{2} }$$

Minimum Detectable Activity (MDA) of the gamma-ray measurement system, especially for a low-level counting system, was evaluated using the following Eq. (3)^10,29^.

3 $${\text{MDA }} = \frac{{K_{\upalpha } \times \sqrt {\upsigma_{{{\text{NB}}}} } }}{{\upvarepsilon_{{\left( {{\text{E}}\upgamma } \right) }} \times {\text{t}}_{{\text{c }}} \times {\text{I}}_{{\upgamma \left( {{\text{E}}\upgamma } \right) }} \times {\text{M}}}}$$

where MDA is in Bq/kg, Kα is the statistical coverage factor, which equals 1.645 at the 95% confidence level^31^, and ${\upsigma }_{{{\text{NB}}}}$ is the standard deviation of the number of counts when a blank sample is measured to determine the background level.

### Assessment of the internal exposure using GIT model

Organ dose calculation requires using mathematical formulas to estimate the energy absorbed by organs and the total nuclear transformations due to radionuclide retention in organs based on specific parameters.

The first parameter, the specific effective energy (SEE) imparted per gram of tissue in the target organ (T) from the emission of a specified radiation R in a source organ (S) per transformation can be determined from the following Eq. (4)^23,32,33^:

4 $${\text{SEE}}_{{\left( {{\text{T }} \leftarrow {\text{ S}}} \right){\text{ R}}}} \equiv \frac{{\sum \left( {{\text{AF}} _{{\left( {{\text{T}} \leftarrow {\text{S}}} \right){\text{R}} }} \times {\text{Y}}_{{\text{R}}} \times {\text{E}}_{{\text{R}}} \times {\text{W}}_{{\text{R}}} } \right) }}{{{\text{M}}_{{\text{T}}} }}\;\left( {\text{MeV/g}} \right)\;{\text{per}}\;{\text{transformation}}$$

where AF_(T ← S) R_: specific absorbed fraction, a radionuclide in organ S emits several kinds of radiation R with yields Y_R_ and average energies E_R_, W_R_: radiation weighting factors for β, γ and x ray W_R_ = 1, for α particles W_R_ = 20^34^, M_T_ mass of the target organ^32^ (Table 1).

**Table 1:** Tissue masses (g) for a reference 70 kg adult male^32^.

Tissue	Mass (g)
Stomach contents	250
Small intestine contents	400
Upper large intestine contents	220
Lower large intestine contents	135
Stomach wall	150
Small intestine wall	640
Upper large intestine wall	210
Lower large intestine wall	160

The second parameter, the cumulative number of transformations U_S_ of a radionuclide into the various sources’ organs over 50 years can be calculated from Eq. (5)^5,17,32,35^:

5 $${\text{U}}_{{\text{S}}} = 8.64 \times 10^{4} \frac{\uptau }{{\text{s}}} \times \left[ { \frac{{ {\text{I}} \frac{\uptau }{{\text{s}}}}}{{\uplambda {\text{e}} \frac{1}{{\text{d}}} }} } \right] \times \left[ {1 - {\text{e}}^{{ - \uplambda {\text{e}} \times {\text{t}}}} } \right]$$

where I is the annual intake of specific radionuclide into S organ which can be determined by multiplying the annual per capita consumption rate of a food product (kg/person/year) by the measured activity concentration of the specific radionuclide (Bq/kg) (Table 2). The effective clearance rate λ_e_ (${\text{d}}^{ - 1}$), represents the radionuclides rate removed from an organ over time, reflecting the radionuclide uptake and retention in body tissue following ingestion^36^, values of λ_e_ are given in Fig. 1.

**Table 2:** The activity concentration of natural radionuclides in beverages (Bq/L) and food (Bq/kg) available in the Egyptian market, the samples number (N0.), and the average annual consumption of food products by adults in Egypt (kg/person/y) based on © Statista 2023 data.

Category	Sample type	Annual intake Kg/y or L/y	No	Activity concentration								
				^226^Ra			^232^Th			^40^K		
				Min	Max	Average	Min	Max	Average	Min	Max	Average
Beverages	Carbonated soft drinks	30.46	12	< 0.13	0.28 ± 0.03	0.22 ± 0.02	< 0.12	< 0.12	< 0.12	< 1.96	8.20 ± 0.33	3.28 ± 0.13
	Energy drinks		2	< 0.13	0.18 ± 0.02	0.16 ± 0.02	< 0.12	0.18 ± 0.01	0.15 ± 0.01	< 1.96	< 1.96	< 1.96
	Tea	2.91	3	< 0.13	0.31 ± 0.03	0.20 ± 0.02	0.13 ± 0.01	0.15 ± 0.02	0.14 ± 0.02	3.22 ± 0.14	5.73 ± 0.24	4.85 ± 0.20
Processed Cereals	Wheat Flour	124	5	< 0.18	0.38 ± 0.02	0.30 ± 0.02	0.18 ± 0.02	0.24 ± 0.02	0.21 ± 0.02	28.74 ± 0.99	89.14 ± 2.92	48.22 ± 1.63
	Bread	36.29	3	0.34 ± 0.02	0.61 ± 0.04	0.44 ± 0.03	0.16 ± 0.02	0.23 ± 0.02	0.20 ± 0.02	35.50 ± 1.23	89.73 ± 2.96	57.11 ± 1.93
	Macaroni	8.16	2	0.19 ± 0.01	0.21 ± 0.02	0.20 ± 0.01	0.13 ± 0.01	0.17 ± 0.01	0.15 ± 0.01	34.79 ± 1.17	35.24 ± 1.19	35.02 ± 1.18
	Instant Noodles	12.97	5	0.19 ± 0.02	0.79 ± 0.07	0.48 ± 0.04	0.14 ± 0.01	0.42 ± 0.04	0.26 ± 0.02	39.46 ± 1.40	61.24 ± 2.10	49.94 ± 1.74
	Corn products	41.11	3	0.38 ± 0.03	0.68 ± 0.05	0.49 ± 0.04	0.17 ± 0.02	0.20 ± 0.02	0.19 ± 0.02	< 2.5	46.53 ± 1.62	26.46 ± 0.93
Milk and Dairy	Milk	22.95	4	< 0.10	0.24 ± 0.02	0.16 ± 0.02	< 0.09	0.11 ± 0.01	0.10 ± 0.01	29.77 ± 1.02	39.49 ± 1.34	36.40 ± 1.34
	Dairy milk (Cheese and yogurt)	1.4	2	0.42 ± 0.03	0.50 ± 0.04	0.46 ± 0.03	< 0.11	0.13 ± 0.01	0.12 ± 0.01	43.99 ± 1.47	49.66 ± 1.67	46.83 ± 1.57
		4.51										
Fish and Meat products	Canned Fishes	2.91	2	0.31 ± 0.03	0.37 ± 0.04	0.34 ± 0.03	0.21 ± 0.02	0.34 ± 0.03	0.28 ± 0.03	33.01 ± 2.61	36.02 ± 1.27	34.52 ± 1.94
	Canned Beef	5.32	2	0.42 ± 0.04	0.77 ± 0.07	0.60 ± 0.06	0.24 ± 0.02	0.30 ± 0.02	0.27 ± 0.03	24.66 ± 0.90	29.55 ± 1.01	27.11 ± 0.95
	Luncheon		2	0.42 ± 0.03	0.68 ± 0.05	0.55 ± 0.04	0.22 ± 0.02	0.24 ± 0.02	0.23 ± 0.02	36.30 ± 1.22	42.37 ± 1.41	39.34 ± 1.32
Table salt	Processed and natural	0.26	4	0.16 ± 0.01	0.32 ± 0.02	0.26 ± 0.02	< 0.09	0.15 ± 0.01	0.11 ± 0.01	5.93 ± 0.22	12.25 ± 0.43	9.31 ± 0.33
All samples			51	< 0.10	0.79 ± 0.07	0.32 ± 0.03	< 0.09	0.42 ± 0.04	0.17 ± 0.02	< 1.96	89.73 ± 2.96	26.45 ± 0.95

The committed equivalent dose to the tissue or organ T for all types of radiation R emitted by specific radionuclides j in organ S at time (t = 50 y) after ingestion of radionuclides is calculated using Eq. (6)^16,37^:

6 $${\text{H}}_{{50\;({\text{T }} \leftarrow {\text{ S}})}} = 1.6 \times 10^{ - 10} \mathop \sum \limits_{{\text{S}}} \mathop \sum \limits_{{\text{j}}} \left[ { U_{{\text{S}}} \times \mathop \sum \limits_{{\text{R}}} {\text{SEE}}_{{\left( {{\text{T}} \leftarrow {\text{S}}} \right){\text{R}}}} } \right]_{{\text{j }}} \quad \left( {{\text{Sv}}} \right)$$

The Effective dose (E), the sum of the weighted equivalent doses in specified tissues and organs of the body over 50 years is calculated using Eq. (7)^5,32,34^:

7 $${\text{E}}_{(50)} = 1.6 \times 10^{ - 10} \times \mathop \sum \limits_{{\text{T}}} W_{{\text{T}}} \times \mathop \sum \limits_{{\text{S}}} \mathop \sum \limits_{{\text{j}}} \left[ { {\text{U}}_{{\text{S}}} \times \mathop \sum \limits_{{\text{R}}} {\text{SEE}}_{{\left( {{\text{T}} \leftarrow {\text{S}}} \right){\text{R}}}} } \right]_{{\text{j }}} \quad \left( {{\text{Sv}}} \right)$$

where w_T_ is the tissue-weighting factor for organ or tissue T^38^.

According to these calculations, the effective doses (μSv/y) received by adults in the four GIT compartments were evaluated by treating each compartment as a target and/or source organ.

## Results and discussion

### Natural radionuclides level in the different categories of foodstuff

The activity concentration levels of natural radionuclides ^226^Ra, ^232^Th, and ^40^K in the studied categories of the most popular foodstuff consumed by Egyptian are given in Table 2. ^40^K exhibited the highest activity concentrations due to its natural abundance and bioaccumulation^39^. Its activity ranged from < 1.96 in carbonated and energy drinks, to 89.73 ± 2.96 Bq/kg in bread. ^226^Ra and ^232^Th levels were generally found in low concentrations. ^226^Ra varied from < 0.10 in milk to 0.79 ± 0.07 Bq/kg in noodles. Similarly, ^232^Th was < 0.09 in milk and table salt samples and had a maximum value of 0.42 ± 0.04 Bq/kg in noodles. In all studied samples ^226^Ra values were higher than that of ^232^Th, and this may be due to the higher solubility and mobility of ^226^Ra compared to ^232^Th^2,40^.

Beverages typically exhibit low activity concentration levels among the studied categories, which may be attributed to their water-based nature and ingredients such as sugar, flavourings, and carbonation, that do not significantly contribute to radioactivity^41^. Carbonated soft drinks have average activity concentrations of 0.22 ± 0.02, < 0.12, and 3.28 ± 0.13 Bq/L, for ^226^Ra, ^232^Th, and ^40^K, respectively. Energy drinks have values of 0.16 ± 0.02 and 0.15 ± 0.01 Bq/L for ^226^Ra and ^232^Th, respectively, while ^40^K was < 1.96 Bq/L. Tea has average values of 0.20 ± 0.02, 0.14 ± 0.02, and 4.85 ± 0.20 Bq/L for ^226^Ra, ^232^Th, and ^40^K, respectively.

As can be seen in Table 3, soft and energy drinks in the present study exhibited higher levels of ^226^Ra than those reported for Tunisia^41^, Hong Kong^42^, and lower that of Malaysia^43^. ^232^Th levels are lower than that reported for Tunisia and Malaysia^41,43^. ^40^K has higher values in the present study compared to that reported for Hong Kong^42^ and lower than that of Mexico^44^. On the other hand, tea samples were found to have lower values of ^226^Ra and ^232^Th and higher values of ^40^K compared to those from Malaysia^43^. These findings suggest that the difference in the radioactivity levels of beverage samples may be attributed to the variations in the raw materials and manufacturing processes.

**Table 3:** The natural radiation levels in foodstuff categories compared to previous literature.

Category	Country	Sample type	Activity concentration (Bq/kg)			References
			^226^Ra	^232^Th	^40^K	
Beverages	Egypt	Carbonated soft drinks	0.22 ± 0.02	< 0.12	3.28 ± 0.13	Present study
		Energy drinks	0.16 ± 0.02	0.15 ± 0.01	< 1.96	
		Tea	0.20 ± 0.02	0.14 ± 0.02	4.85 ± 0.20	
	Tunisia	Carbonated soft drinks	≤ 0.18	≤ 0.13	≤ 4.51	^41^
	Hong Kong	Soft drinks	< 0.002–0.015	NA	0.056–0.22	^42^
	Mexico	Soft drinks	NA	NA	4.0–35	^44^
	Malaysia	Carbonated drinks	0.25 ± 0.001	1.06 ± 0.001	2.35 ± 0.001	^43^
		Energy drinks	0.55 ± 0.001	0.40 ± 0.001	5.07 ± 0.001	
		Tea	0.50 ± 0.001	0.22 ± 0.001	0.11 ± 0.001	
Processed Cereals	Egypt	Wheat-flour	0.30 ± 0.02	0.21 ± 0.02	48.22 ± 1.63	Present study
		Bread	0.44 ± 0.03	0.20 ± 0.02	57.11 ± 1.93	
		Macaroni	0.20 ± 0.01	0.15 ± 0.01	35.02 ± 1.18	
		Macaroni	NA	NA	36	^54^
	Brazilian	Flours, Pasta and Bakery	0.25 ± 0.07	0.010 ± 0.004	24 ± 4	^2^
	Korea	Bread	NA	NA	33 ± 9 (22–51)	^50^
	Swedish	Bread	NA	NA	72 ± 4	^45^
		Wheat-flour	NA	NA	67 ± 6	
		Macaroni	NA	NA	81 ± 2	
	Turkey	Wheat-flour	10.80 ± 2.37	11.00 ± 2.23	274.10 ± 25.5	^8^
		Bread	17.66 ± 4.31	22.86 ± 3.83	166.46 ± 30.6	
		Macaroni	13.18 ± 3.85	18.45 ± 4.24	105.67 ± 29.8	
	Egypt	Instant noodles	0.48 ± 0.04	0.26 ± 0.02	49.94 ± 1.74	Present study
	Iraq		2.38–31.92	1.51–9.27	113.1–392.5	^46^
	Hong Kong		< 0.03	NA	29.55–64.54	^42^
	Egypt	Corn	0.41 ± 0.03	0.17 ± 0.02	30.74 ± 1.08	Present study
		Corn Flour	0.68 ± 0.05	0.20 ± 0.02	46.53 ± 1.62	
		Corn	0.59 ± 0.28	0.55 ± 0.43	NA	^47^
	Iran	Corn	0.81 ± 0.03	0.85 ± 0.03	101.52 ± 1.29	^48^
	Italy	Corn Flour	NA	NA	99 ± 19	^64^
Milk and Dairy	Egypt	milk	0.16 ± 0.02	0.10 ± 0.01	36.40 ± 1.34	Present study
		Dairy milk	0.46 ± 0.03	0.12 ± 0.01	46.83 ± 1.57	
		Milk	0.033	0.0001	21	^26^
	Korea	Milk	NA	NA	43 ± 8 (18–48)	^50^
	Italian		NA	NA	38 ± 5	^52^
	Netherlands		NA	NA	53.7 ± 11.1	^53^
	Lebanon	Milk	NA	NA	39.1	^55^
		Dairy products	NA	NA	43.7	
	Egypt	Cheese	NA	NA	37–58	^54^
	Swedish	Cheese and yoghurt	NA	NA	28 ± 1–58 ± 1	^45^
	Brazilian	Dairy products	0.065 ± 0.03	< 0.010	49 ± 10–52 ± 11	^2^
Fish and meat products	Egypt	Canned fish	0.34 ± 0.03	0.28 ± 0.03	34.52 ± 1.94	Present study
	Jordan	Canned tuna and sardine	< 0.03–1.80	< 0.03–1.3	11–260	^56^
	Nigeria	Canned tuna and mackerel	17–24	8–17	59–64	^58^
	Iraq	Canned tuna and sardine	NA	3.83–7.98	NA	^59^
	Kuwait	Canned seafood	0.4–2	BDL	5–42	^57^
	Egypt	Canned beef	0.60 ± 0.06	0.27 ± 0.03	27.11 ± 0.95	Present study
		Beef	0.08–0.12	0.002–0.003	18.07–20.67	^61^
	Nigeria	Canned beef	14 ± 4	14 ± 4	50 ± 14	^58^
	Turkey	Beef	BDL	0.32 ± 0.16	52.17 ± 5.70	^60^
Table salt	Egypt	Processed and natural salt	0.26 ± 0.02	0.11 ± 0.01	9.31 ± 0.33	Present study
		Processed and natural salt	0.46–32.6	0.20–10.5	0.42–158.6	^63^
	Namibia	Processed salt	2.17 ± 0.19	0.20 ± 0.02	2.28 ± 0.39	^65^
	Jordan	Dead sea cooking salt	1.18 ± 0.12	0.51 ± 0.07	6.96 ± 0.87	^64^
	Brazilian	Processed salt	0.03–0.05	< 0.010	96–108	^2^

*NA* not applicable, *BDL* below detection limit.

Wheat and corn are vital food crops worldwide. In Egypt, bread is a staple of the diet. In processed cereals, ^226^Ra, ^232^Th, and ^40^K activity concentrations had average values of 0.30 ± 0.02, 0.21 ± 0.02, and 48.22 ± 1.63 Bq/kg in wheat flour; 0.44 ± 0.03, 0.20 ± 0.02, and 57.11 ± 1.93 Bq/kg in bread; 0.20 ± 0.01, 0.15 ± 0.01, and 35.01 ± 1.18 Bq/kg in macaroni; 0.48 ± 0.04, 0.26 ± 0.02, and 49.94 ± 1.74 Bq/kg in instant noodles; and 0.49 ± 0.04, 0.19 ± 0.02, and 26.46 ± 0.93 Bq/kg in corn products. The highest activity concentration of ^226^Ra and ^232^Th (0.79 ± 0.07 and 0.42 ± 0.04 Bq/kg, respectively) was found in instant noodles, while the highest activity concentration of ^40^K (89.73 ± 2.96 Bq/kg) was found in bread. Higher levels of ^40^K were observed in bread, instant noodles, and wheat flour, whereas macaroni and corn products showed lower values, The variation in the radioactivity level of processed cereals can be attributed to the type of cereals used, the environment in which it was grown, and processing techniques.

The data available in the literature (Table 3) show that the average activity concentration of ^226^Ra, ^232^Th, and ^40^K in the processed cereals under study was lower than their counterparts in Turkey and Sweden^8,45^ (wheat flour, bread, and macaroni), Iraq^46^ (instant noodles), Egypt, Iran, and Italy^47–49^ (corn and corn flour). While it was higher than their counterparts in Brazil and Korea^2,50^ (flour, bread, and macaroni), and Hong Kong^42^ (instant noodles).

The average values for ^226^Ra, ^232^Th, and ^40^K in milk and dairy samples were 0.16 ± 0.02, 0.10 ± 0.01, and 36.40 ± 1.34 Bq/L in milk, and 0.46 ± 0.03, 0.12 ± 0.01, and 46.83 ± 1.57 Bq/kg in dairy products (cheese and yoghurt), respectively. These results indicated that the radioactivity level in dairy products is slightly higher than that in milk, which may be due to their nutritional content as they contain saturated vegetable fats and fiber^51^.

In comparison with the results of a previous study in Egypt^26^, it is clear that ^226^Ra, ^232^Th and ^40^K concentrations in the milk samples under study were higher than those recorded in the previous study. On the other hand, the ^40^K concentration of milk samples in the present study was lower than that observed in other countries such as Korea^50^, Italy^52^, and Netherland^53^. ^40^K concentration in the dairy products under study showed a lower level compared to that observed in Sweden^45^, Brazil^2^, and Egypt^54^ and higher than that in Lebanon^55^. Meanwhile, ^226^Ra was lower, and ^232^Th concentration was higher than that reported for Brazilian dairy products^2^ (Table 3).

The analyzed fish and meat products showed the average levels of ^226^Ra, ^232^Th, and ^40^K: in canned fish, 0.34 ± 0.03, 0.28 ± 0.03, and 34.52 ± 1.94 Bq/kg; in canned beef, 0.59 ± 0.06, 0.27 ± 0.03, and 27.11 ± 0.95 Bq/kg; and in luncheon, 0.55 ± 0.04, 0.23 ± 0.02, and 39.34 ± 1.32 Bq/kg, respectively. The results show that the highest ^226^Ra concentrations were recorded in canned beef, while the highest ^232^Th concentrations were in canned fish and the highest ^40^K concentrations were in luncheon. The activity concentration levels in canned fish are within the range of values recorded in Jordan^56^ and Kuwait^57^ and below those recorded in Nigeria and Iraq^58,59^. It is worth noting that fish type, brine/vegetable oil compositions used for storage, and the marine ecosystems of the origin country affect radioactivity levels of canned fish^56^. Values of activity concentrations of canned beef in the current study were lower than those of Nigeria and Turkey^58,60^ but higher those that recorded in a previous Egyptian study^61^ (Table 3).

Salt (NaCl) is commonly used for seasoning and food preservation, typically extracted from marine sediments with low radionuclide levels as it is a mineral that does not promote radionuclide accumulation, unlike plants^62^. Measurements of natural radioactivity levels in table salt samples indicate average values of 0.26 ± 0.02, 0.11 ± 0.01, and 9.31 ± 0.33 Bq/kg for ^226^Ra, ^232^Th, and ^40^K, respectively. Radioactivity levels in analyzed salt samples are lower than that observed in a previous Egyptian study^63^, whereas, ^40^K value is higher than that recorded in Jordan and Namibia salt^64,65^ (Table 3).

### Assessing the gastrointestinal tract GIT’s effective dose

The effective dose (μSv/y) received by the different compartments of the adult gastrointestinal tract (stomach, small intestine, upper large intestine, and lower large intestine) due to the intake of the studied foods was calculated based on Eqs. (4–7) and the results obtained are shown in Table 4. Wheat flour was excluded from the dose calculations as it is usually consumed as bread or macaroni.

**Table 4:** The average adult’s effective doses received by GIT compartments (μSv/y) resulting from the intake of studied samples.

Category	Sample type	E (St → St)*	E (St → Stw)*	E (SI → SI)*	E (SI → SIw)*	E (SI → BF)*	E (ULI → ULI)*	E (ULI → ULIw)*	E (LLI → LLI)*	E (LLI → LLIw)*
		Stomach content	Stomach Wall	Small intestine content	Small intestine Wall	Body fluids	Upper large intestine content	Upper large intestine wall	Lower large intestine content	Lower large intestine wall
Beverages	Carbonated soft drinks	0.266	0.444	0.543	0.340	1.55E−05	3.29	3.45	9.66	8.15
	Energy drinks	0.231	0.385	0.493	0.308	9.26E−06	2.99	3.13	8.77	7.40
	Tea	0.010	0.017	0.021	0.013	8.72E−07	0.13	0.13	0.37	0.32
Processed Cereals	Bread	0.866	1.453	1.223	0.766	3.21E−04	7.43	7.77	21.80	18.40
	Macaroni	0.110	0.184	0.152	0.095	4.43E−05	0.92	0.96	2.69	2.27
	Instant Noodles	0.328	0.550	0.511	0.319	1.00E−04	3.09	3.24	9.08	7.66
	Corn products	0.852	1.428	1.484	0.927	1.69E−04	8.98	9.42	26.35	22.25
Milk and Dairy	Milk	0.260	0.438	0.309	0.193	1.29E−04	1.87	1.96	5.50	4.64
	Dairy milk	0.063	0.106	0.093	0.058	2.07E−05	0.56	0.59	1.64	1.39
Fish and Meet products	Canned fishes	0.057	0.095	0.094	0.059	1.56E−05	0.57	0.60	1.68	1.42
	Canned beef	0.135	0.225	0.243	0.152	2.24E−05	1.48	1.55	4.33	3.65
	Luncheon	0.133	0.222	0.220	0.137	3.24E−05	1.34	1.40	3.90	3.29
Table salt	Processed and natural	0.003	0.005	0.005	0.003	3.75E−07	0.03	0.03	0.09	0.08
All samples		3.31	5.55	5.39	3.37	8.82E−04	32.67	34.23	95.85	80.90

* Source organ → Target organ.

#### Stomach content doses

The doses received by stomach content due to food intake were calculated as mentioned above and considering the stomach as the source and target organ. For beverages group, the lowest dose to the stomach content was from tea, at 0.010 μSv/y, and the highest from carbonated soft drinks, at 0.266 μSv/y, possibly due to the higher consumption rate of soft drinks compared to tea. For processed cereals, the lowest stomach content dose was from macaroni (0.110 μSv/y) and the highest from bread (0.866 μSv/y), probably due to the higher consumption rate and higher radioactivity level of bread compared to macaroni (see Table 2). For milk and dairy products, although the level of radioactivity in milk was lower than that in dairy products, the dose from milk was higher than that from dairy products, being 0.260 and 0.063 μSv/y, respectively, which may be attributed to the higher consumption rate of milk compared to dairy products. In fish and meat products, canned beef and luncheon had higher doses than canned fish (0.135, 0.133, and 0.057 μSv/y, respectively, probably due to the higher consumption rate and higher radioactivity level of canned beef and luncheon compared to canned fish (Table 2). The dose from table salt was the lowest dose among the foods studied, at 0.003 μSv/y. The reason for this is the low consumption rates of table salt.

#### Stomach wall doses

Stomach wall doses were computed considering the stomach as the source organ and the stomach wall as the target organ, displaying a similar trend to stomach content doses in terms of the varying doses from different food types. Among beverages, tea exhibited the lowest dose (0.017 μSv/y) while soft drinks showed the highest dose (0.444 μSv/y). Within processed cereals, macaroni had the lowest dose (0.184 μSv/y) and bread the highest (1.453 μSv/y). In milk and dairy products, milk had the highest dose from its products (0.438 and 0.106 μSv/y, respectively). Canned fish represented the lowest dose in meat and fish products compared to canned beef and luncheon (0.095, 0.225, and 0.222 μSv/y, respectively). Table salt maintained the lowest dose across all studied samples (0.005 μSv/y). However, stomach wall dose values were elevated by 67% compared to stomach content. This variance could be attributed to the smaller mass of the stomach wall (150 g) in contrast to the stomach content mass (250 g), potentially resulting in a higher specific effective energy (SEE) delivered per gram of tissue in the target organ and consequently a higher dose.

#### Small intestine content doses

The doses assigned to the small intestine content were computed with the small intestine content acting as both the source and target organ. Among beverages, tea exhibited the lowest dose at 0.021 μSv/y, while soft drinks showed the highest dose at 0.543 μSv/y. In the category of processed cereals, macaroni had the lowest dose (0.152 μSv/y) and corn products the highest (1.484 μSv/y). Regarding milk and dairy products, milk had a higher dose compared to dairy products (0.309 and 0.093 μSv/y, respectively). Within fish and meat products, canned beef and luncheon showed higher doses than canned fish (0.243, 0.220, and 0.094 μSv/y, respectively). Table salt represented the lowest dose among the foods examined, at 0.005 μSv/y.

#### Small intestine wall doses

Small intestine wall doses were conducted with the small intestine content serving as the source organ and the small intestine wall as the target organ, and the results show a pattern akin to the doses observed for small intestine content across different food types. Generally, small intestine wall dose values were approximately 37.5% lower than those for small intestine content. This could be linked to the greater mass of the small intestine wall (640 g) in comparison to the mass of the small intestine content (400 g), potentially resulting in a reduced specific effective energy (SEE) delivered per gram of tissue within the target organ and thus leading to a lower overall dose.

#### Body fluids dose

Radionuclides are absorbed through the SI wall, diffuse into blood vessels, and transfer through body fluids (BF) to other organs of the human body^24^. The metabolic rate constant λ_B_ (Fig. 1) indicates the activity transfer rate from the SI to body fluids^5^, which estimated by f_1_, the fraction of element reaching body fluids post-ingestion. f_1_ values of ^226^Ra, ^232^Th, and ^40^K in the metabolic data of the ICRP were used^38^. The results of the effective dose of (BF) received due to food intake showed that, the lowest dose due to beverage intake was for tea at 8.72E−07 μSv/y, and the highest for soft drinks at 1.55E−05 μSv/y. For processed cereals intake macaroni had the lowest dose at 4.43E−05 μSv/y, and bread had the highest at 3.21E−04 μSv/y. Milk intake causes higher dose (1.29E−04 μSv/y) than dairy products (2.07E−05 μSv/y). The dose from luncheon intake (3.24E−05 μSv/y) is higher than canned fish (1.56E−05 μSv/y). Also, table salt displayed the lowest dose at 3.75E−07 Sv/y. These results reflect that the dose values transmitted through the blood are minimal, as absorbed radionuclides undergo distribution and dilution in the circulatory system^11^.

#### Upper large intestine content doses

The doses assigned to the upper large intestine content yielded comparable findings to those of the small intestine but with elevated values. Among beverages, tea had the smallest dose of 0.13 μSv/y, while the highest dose of 3.29 μSv/y for soft drinks. In the processed cereals, macaroni recorded the lowest dose of 0.92 μSv/y and corn products the highest of 8.98 μSv/y. In milk and dairy products, milk showcased a higher dose in contrast to dairy products (1.87 and 0.56 μSv/y, respectively). Within fish and meat products, canned beef and luncheon displayed higher doses than canned fish (1.48, 1.34, and 0.57 μSv/y, respectively). Table salt exhibited the lowest dose among the scrutinized foods, 0.03 μSv/y.

#### Upper large intestine wall doses

The upper large intestine wall dose values were 5% higher than the upper large intestine content values, with the same dose pattern observed for the upper large intestine content of the different food types. As mentioned above, the reason may stem from the disparity in mass between organ content and organ wall (as illustrated in Table 1).

#### Lower large intestine content doses

The lower large intestine content doses parallel those of the upper large intestine but with increased values. Tea had the lowest dose at 0.37 μSv/y, while soft drinks had the highest at 9.66 μSv/y in the beverages category. Macaroni had the lowest dose in processed cereals at 2.69 μSv/y, while corn products had the highest at 26.35 μSv/y. Milk had a higher dose than dairy products (5.50 μSv/y compared to 1.64 μSv/y). Canned beef had the highest dose in fish and meat products at 4.33 μSv/y, while canned fish had the lowest at 1.68 μSv/y. Table salt exhibited the lowest dose among the foods studied, at 0.09 μSv/y.

#### Lower large intestine wall doses

The lower large intestine wall doses were 15.6% less than the lower large intestine content doses, with a consistent dosage pattern observed across different food types for the lower large intestine content.

**Fig. 2:**
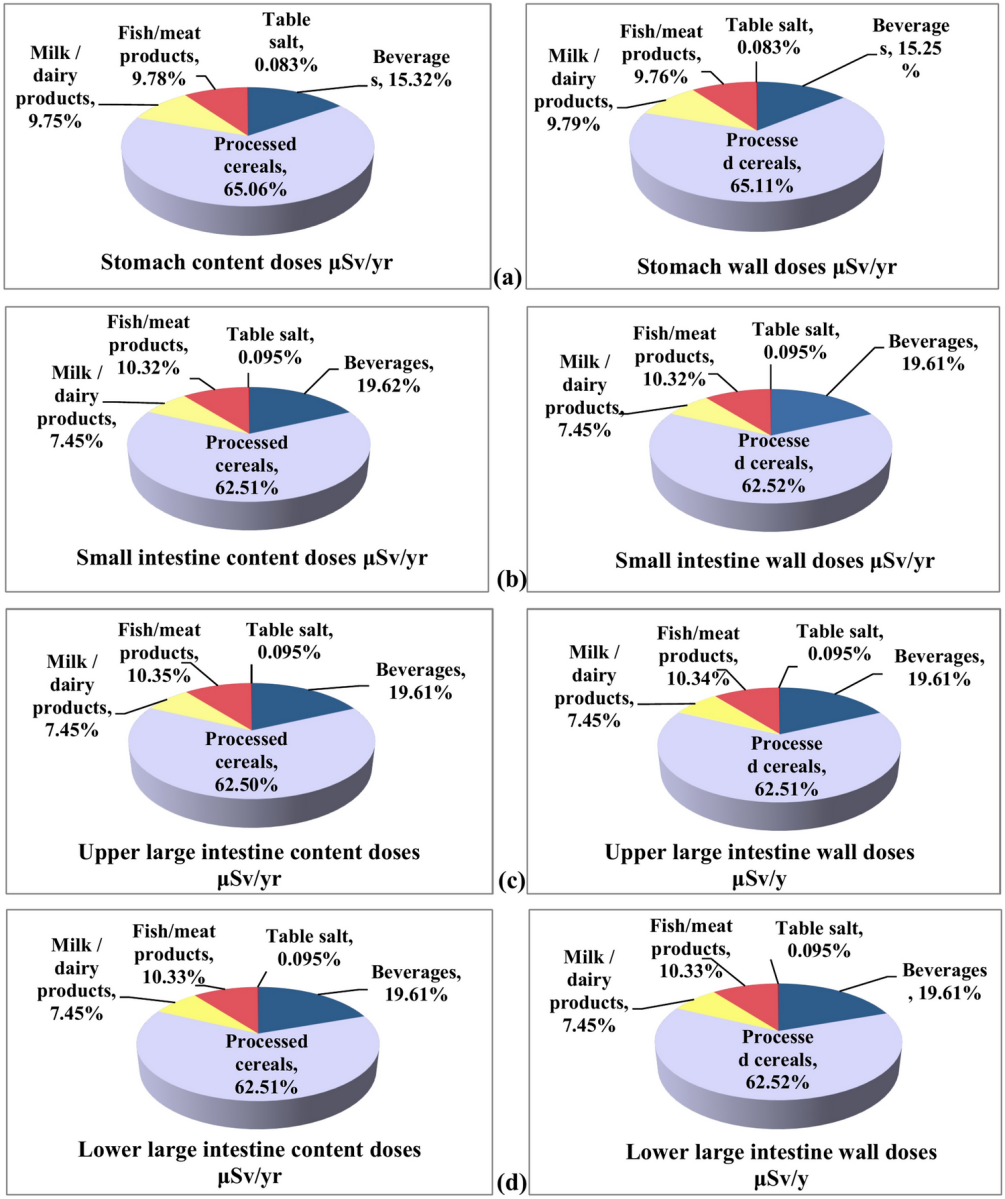
Food groups contributions to the effective dose (μSv/y) received by (a) Stomach, (b) Small intestine, (c) Upper large intestine, and (d) Lower large intestine.

Figure 2 shows the contributions of the studied food groups to the doses received by various compartments of the adult gastrointestinal tract, indicating that doses ranked as follows: processed cereals > beverages > fish/meat products > milk/dairy products > table salt. Additionally, the dose from beverages rose from 15.3% in the stomach to 19.6% in the other compartments of the gastrointestinal tract, while the dose from milk/dairy products decreased from 9.8% to 7.5% and processed cereals from 65.1% to 62.5%.

**Fig. 3:**
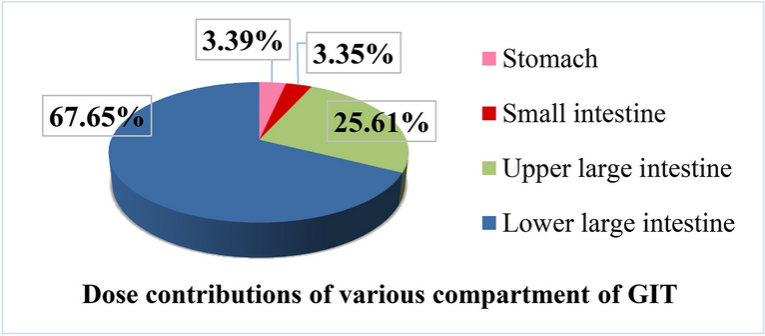
Dose contributions of various compartment of GIT.

In Fig. 3, the gastrointestinal tract dose ratios from the studied food reveal that the stomach and small intestine receive a minimal amount (3%), while the lower large intestine (LLI) accounts for the largest share (68%). This could be due to the prolonged metabolic residence in the LLI compartment (up to 24 h) increases the absorption likelihood of food components, including radionuclides, through both LLI walls and contents^5,32,66^. Also, discrepancies in organ masses lead to dose value differences.

The findings from this study indicate that the effective doses (μSv/y) received by each compartment of the adult gastrointestinal tract, (stomach: 8.86, small intestine: 8.76, upper large intestine 66.90, lower large intestine 176.76) or the entire digestive system (261.28), from the consumption of the examined foods, fall below the safe thresholds set by global organizations. The United Nations Scientific Committee on the Effects of Atomic Radiation (UNSCEAR) has established a safe limit of 290 μSv/y^66^, while the World Health Organization (WHO) has outlined a safe range between 250 and 400 μSv/year for food intake^67^. Therefore, there are no significant radiation risks associated with the consumption of the investigated food items.

## Conclusion

Gamma-ray spectrometry was used to analyze natural radionuclide levels (^226^Ra, ^232^Th, and ^40^K) in various foods people frequently consume in Egypt. ^40^K had the highest activity concentrations at 89.73 ± 2.96 Bq/kg in bread. Levels of ^226^Ra and ^232^Th were generally lower, with the highest values of 0.79 ± 0.07 Bq/kg and 0.42 ± 0.04 Bq/kg in noodle samples, respectively. Furthermore, the doses due to the ingestion of analyzed foods received by different compartments of the adult gastrointestinal tract were evaluated mathematically using a GIT model. The contributions of the studied foods to the doses ranked as follows: processed grains > beverages > fish/meat products > milk/dairy products > table salt. Among the different compartments of the gastrointestinal tract, LLI received the highest dose of 176.76 μSv/y which represents 68% of the total dose received by the entire gastrointestinal tract (261.28 μSv/y). The results showed that the dose transmitted through the blood due to the intake of food under study are minimal compared to the doses received by the digestive system. Overall, the effective doses received by GIT compartments from all studied foodstuffs were below the limits set by UNSCEAR, and WHO, confirming the radiological safety of these foodstuff items. However, continuous monitoring remains essential to ensure consumer safety.

## Data Availability

All data generated or analyzed during this study are included in this article. The raw data supporting the conclusions of this article will be made available by the corresponding author, [Yasmine Abdalbasit Abbas], at [Yasmine.abdalbasit@sci.svu.edu.eg], without undue reservation.
